# A Survey of the Perception of Comprehensiveness among Dentists in a Large Brazilian City

**DOI:** 10.3390/ijerph110404249

**Published:** 2014-04-16

**Authors:** Grazielle C. M. Mattos, Cleiton G. Sirineu, Bruno R. Teixeira, Jennifer E. Gallagher, Saul M. Paiva, Mauro H. N. G. Abreu

**Affiliations:** 1 Department of Community and Preventive Dentistry, Universidade Federal of Minas Gerais, Belo Horizonte, Av. Antônio Carlos, 6627 Belo Horizonte, Minas Gerais 31270901, Brazil; E-Mails: gueziabh@yahoo.com.br (G.C.M.M.); cleitonsirineu@hotmail.com (C.G.S.); rafa_tx@hotmail.com (B.R.T.); 2 Department of Dental Public Health, King’s College London, Room 220, Dental Institute, Denmark Hill, London SE5 9RS UK; E-Mail: jenny.gallagher@kcl.ac.uk; 3 Department of Orthodontics and Paediatric Dentistry, Universidade Federal of Minas Gerais, Belo Horizonte, Av. Antônio Carlos, 6627 Belo Horizonte, Minas Gerais 31270901, Brazil; E-Mail: smpaiva@uol.com.br

**Keywords:** comprehensive health care, oral health, primary health care, health personnel

## Abstract

*Objectives:* To quantitatively identify the perception of dentists regarding comprehensiveness and its domains of “patient welcoming”, “bonding” and “quality of care” in primary dental care settings of a large Brazilian city. *Methods:* A questionnaire was administered to all dentists comprising the primary health care service to Belo Horizonte with tenured jobs and 40 work hours per week, totalling a population of 207 professionals. The response rate was 90.34%. A pilot test was conducted with 44 dentists working in primary care for at least two years and who did not participate in the main study. Descriptive statistical analysis involved calculating proportions. No confidence intervals were calculated because this was a census study. *Results:* In most items (79.0%), professionals’ perceptions about the comprehensiveness were overwhelmingly positive. When we stratified the analysis by domain and checked those items about which dentists had a less favourable perception, 22.7% were in the patient welcoming domain, 25.0% were in the bonding domain and 12.5% were in quality of care. *Conclusions:* Comprehensiveness, as an approach in health care practice, needs to be enhanced, and there is evidence that these dentists are aware of its importance.

## 1. Introduction

Many public health systems around the world are attempting to establish guidelines to improve the performance of health services. To achieve this, they are adopting Primary Health Care (PHC) as a base to focus on prevention and health promotion actions [1,2,3]. PHC is based on a group of aspects that when connected are equally important to structure an efficient healthcare system and reduce health inequalities, considering social, economic and cultural contexts [1,3,4,5]. The main aspects, such as universal health access, continuity of care and comprehensiveness, are considered paramount in this structure in order to enhance the quality of care provided [3,6].

Universal health access calls for making healthcare systems functional and removing supply-side barriers; it is an approach that considers care without obstacles. Continuity of care is the relationship built between the patient and health professional over time, through patient welcoming and bonding with the service, and seeks to respond to the health needs of the population [1,5,6]. Comprehensiveness seeks to take care of people as whole beings in the circumstances in which they live and provide all of the care that people might need; it implies a comprehensive approach to individuals in which the full range of their health needs is recognised. It is a two-dimensional concept, so it also seeks to ensure that more health services at all care levels are interconnected and provided, from prevention to tertiary care [3,6,7].

The focus of our study is on the comprehensiveness approach and its understanding by health professionals, specifically dentists. One meaning of comprehensiveness is related to a way of organising health practices that seeks multidisciplinary and team care. It is necessary that professionals who implement the practices understand and are included in this process because on the basis of comprehensiveness, the health services are organised by making a link between programmed and spontaneous flows of patients, taking advantage of the opportunities generated for the application of diagnostic protocols and identification of risk situations for health, as well as the development of sets of health promotion actions in the community [8,9]. 

To understand planning and management in health systems, the contribution of the healthcare workforce is increasingly crucial. The availability of a mix of healthcare occupations across various settings and quantitative, methodical analyses of the stock are essential to motivating better understanding of human resources issues in health care and to identifying problems and solving issues in this area. The necessity to reinforce more studies is overriding, and engaging the healthcare workforce that can potentially produce relevant data that are often underused in health research is very important in this process. Different general data sets can be used as instruments for making assessments of human resources in healthcare, such as sample questionnaires and routine administrative records [10,11,12].

The aim of this study was to quantitatively identify the perception of dentists about comprehensiveness and its domains of “patient welcoming”, *i.e.*, the act of receiving the patient in the clinic and giving a response to their problem; “bonding”, *i.e.*, the link between the patient and the health service; and “quality of care” in dental PHC settings of a large Brazilian city. These concepts are fundamental to exploring the nature of the comprehensiveness approach [1,13,14,15]. It is important for the comprehensiveness element to be reinforced in the critical awareness of health professionals and the population to enable the achievement of innovative, integrative and mutual actions in the healthcare system [7,9,10,11,12,13].

## 2. Methods

This study is part of the second phase of a larger research programme, currently in progress, that proposes to develop an instrument to assess primary care from the dentists’ perspective in domains related to comprehensiveness of care. Parallel studies are examining patient perspectives.

In the first phase, the concept of comprehensiveness was analysed qualitatively through the focus group technique to identify variables and items that should be included in the instrument. In the second phase, the quantitative validation process was carried out, which consists of several steps, one of which was the application of the instrument.

**Figure 1:**
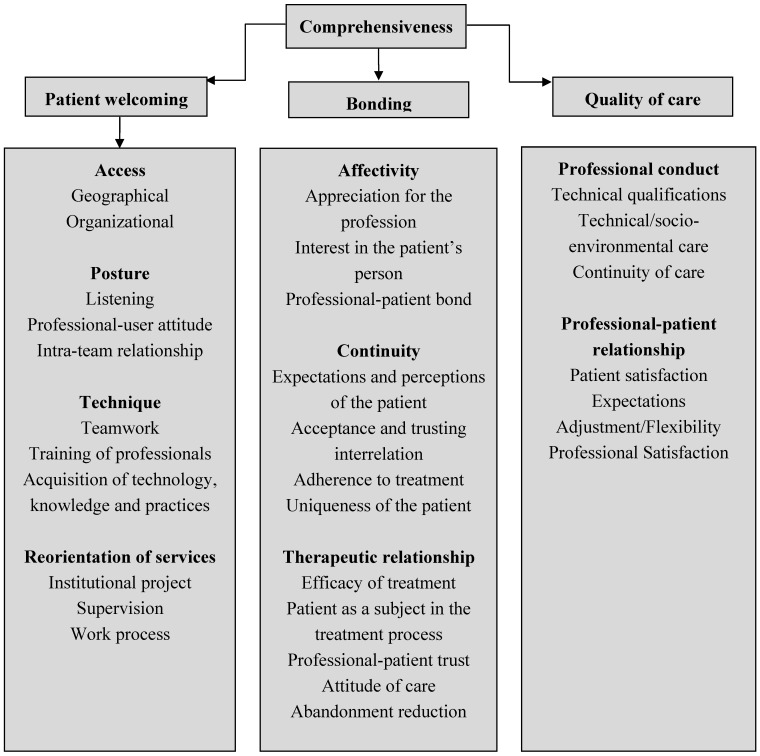
Domains and aspects related to comprehensiveness to be addressed in the assessment of oral health in PHC.

Note: Source: Adapted from Silva Júnior; Mascarenhas [13].

In the initial phase of the research, three key domains in the definition of comprehensiveness of care were discussed: patient welcoming, bonding and quality of care [13]. Figure 1 illustrates how these domains and their aspects propose a structural link between the concepts of PHC as a way to assess the daily routine of services, professional practices and their relationship with the population [2,6,9,13]. 

The research took place in the city of Belo Horizonte, Brazil. It is the sixth largest city in the country, with approximately 2,450,000 inhabitants and an area of 330 km^2^. The local health system has a historic tradition in the development of primary care and seeks to structure its system toward the integration of actions, which involves the Medical Specialities Centre and the Centre for Dental Specialities. At the time of the study, the primary health care for dental care consisted of 264 teams divided into 147 health centres in the city [16]. 

A pilot test was conducted with 44 dentists working in primary care for at least two years and who did not participate in the main study. To verify the performance and reliability of the instrument, the test/retest method was employed. The questionnaire was administered twice in the same individuals with an interval of 10–14 days. The weighted Kappa was greater than 0.60.

Data collection was conducted from July to September 2012. The instrument was administered to all dentists in primary health care service in Belo Horizonte with tenured jobs and 40 work hours per week, totalling a population of 207 professionals. The distribution of questionnaires to the 207 dentists took place with the collaboration of the staff from the City Hall. All envelopes were addressed to the healthcare clinics via interoffice mail, and the local managers from each clinic were instructed to remind the participants and to collect the questionnaires in three working days. The response rate was 90.34%.

The database was constructed in the programme EpiData version 3.1 (EpiData Association, Odense M, Denmark). The data were entered twice by different researchers and subsequently validated. This database was exported to SPSS version 20.0 (SPSS Inc., Chicago, IL, USA). At the end of this phase and after execution of the initial stages of the validation process (data not shown), the instrument was constructed with 38 items (Kaiser-Meyer-Olkin measure equal to 0.62 and Cronbach’s alpha equal to 0.76) designed to assess the dentists’ perceptions about dental care in primary care and about comprehensiveness of care in the actions developed in the health service. The questionnaire used an ordinal scale with five response options: always, almost always, sometimes, rarely and never. There were additional options: do not know, refuse to respond and not applicable. The instrument consisted of 38 items (Table 1), with the first 22 about the patient welcoming domain, the following eight relating to bonding and the last eight to the quality of care. Descriptive statistical analysis involved calculating proportions. For each question, the perception was considered positive when more than 50% of the dentists responded with the options “always” and “almost always”. No confidence intervals were calculated because this was a census study. The study was submitted to and approved by the Ethics Committee for Human Research of the Universidade Federal de Minas and the city of Belo Horizonte (protocol number 0437.0.203.410-11A.). Each dentist signed an informed consent after being informed about the objectives of the research.

**Table 1:** Items of the instrument and frequency of responses.

Items	Always	Almost Always	Sometimes	Rarely	Never	Do not Know/Refuse to Respond/Not Applicable
1—Easy access to dental treatment	104	72	11	–	–	–
	(55.6%)	(38.5%)	(5.9%)			
2—Easy access to emergency dental care	157	28	2	–	–	–
	(84.0)	(15.0%)	(1.1%)			
3—The population have access to oral health care through patient welcoming	14	38	7	–	–	–
	(75.9%)	(20.3%)	(3.7%)			
4—Patient welcoming for oral health care daily	164	12	4	–	7	–
	(87.7%)	(6.4%)	(2.1%)		(3.7%)	
5—Patient welcoming performed by dentist	77	23	54	20	13	–
	(41.2%)	(12.3%)	(28.9%)	(10.7%)	(7.0%)	
6—Using protocol for the classification of risk and the priority of health care service as part of patient welcoming	101	37	19	7	20	3
	(54.0%)	(19.8%)	(10.2%)	(3.7%)	(10.7%)	(1.6%)
7—The population have access to oral health care by appointment	155	28	4	–	–	–
	(82.9%)	(15.0%)	(2.1%)			
8—While waiting, the patient participates in preventive and/or educational activities in oral health care	28	30	48	46	34	1
	(15.0%)	(16.0%)	(25.7%)	(24.6%)	(18.2%)	(0.5%)
9—Friendly relationship with patients	144	42	1	–	–	–
	(77.0%)	(22.5%)	(0.5%)			
10—Enough dedicated time to listen to patients’ complaints.	124	59	4	–	–	–
	(66.3%)	(31.6%)	(2.1%)			
11—Clear communication between the dentist and the patient	125	58	4	–	–	–
	(66.8%)	(31.0%)	(2.1%)			
12—Investigations concerning the patient’s life such as work, leisure and housing during the appointment	36	61	77	11	–	2
	(19.3%)	(32.6%)	(41.2%)	(5.9%)		(1.1%)
13—Knowledge of oral health care team about the oral health problems of the population of the region	56	96	31	2	2	–
	(29.9%)	(51.3%)	(16.6%)	(1.1%)	(1.1%)	
14—Knowledge of other health care professionals about oral health care problems of the population of the region	13	51	82	28	5	7
	(7.0%)	(27.3%)	(43.9%)	(15.0%)	(2.7%)	(3.7%)
15—Systematic home visits by the oral health care team	22	44	69	50	2	–
	(11.8%)	(23.5%)	(36.9%)	(26.7%)	(1.1%)	
16—Resolution, at the level of primary care, of the oral health needs of the patient	122	62	3	–	–	–
	(65.2%)	(33.2%)	(1.6%)			
17—Participation of the oral health care team in planning meetings with other professionals from the family health care team	74	62	41	7	3	–
	(39.6%)	(33.2%)	(21.9)	(3.7%)	(1.6%)	
18—Development of activities together by the oral health care team and other professionals on the family health care team	51	62	66	6	2	–
	(27.3%)	(33.2%)	(35.3%)	(3.2%)	(1.1%)	
19—Planning patient care with the aid of other health professionals	40	40	76	22	9	–
	(21.4%)	(21.4%)	(40.6%)	(11.8%)	(4.8%)	
20—Execution of the work supported by the service management	91	57	32	2	1	4
	(48.7%)	(30.5%)	(17.1%)	(1.1%)	(0.5%)	(2.1%)
21—Training of auxiliary staff to conduct promotion and prevention actions in oral health by the dentist	38	35	74	33	6	1
	(20.3%)	(18.7%)	(39.6%)	(17.6%)	(3.2%)	(0.5%)
22—Providing training course to the dentist that includes family health care	45	50	63	23	5	1
	(24.1%)	(26.7%)	(33.7%)	(12.3%)	(2.7%)	(0.5%)
23—Confidence in performing procedures included in primary oral health care	89	87	8	1	–	2
	(47.6%)	(46.5%)	(4.6%)	(0.5%)		(1.0%)
24—Focus on the oral health of patients	158	21	7	1	–	–
	(84.5%)	(11.2%)	(3.7%)	(0.5%)		
25—Knowledge of each patient’s medical records	71	89	19	6	1	1
	(38%)	(47.6%)	(10.2%)	(3.2%)	(0.5%)	(0.5%)
26—Realisation of referring patients to specialised care	117	8	2	–	–	–
	(94.7%)	(4.3%)	(1.1%)			
27—Transmission of information regarding patient's oral health to the specialist	114	49	14	7	3	–
	(61.0%)	(26.2%)	(7.5%)	(3.7%)	(1.6%)	
28—Return of the patient from specialised care with a written referral prepared by the specialist	14	69	77	24	3	–
	(7.5%)	(36.9%)	(41.2%)	(12.8%)	(1.6%)	
29—Permission for continuity of care in the flow between primary and specialised care	17	71	83	16	–	–
	(9.1%)	(38.0%)	(44.4%)	(8.6%)		
30—Patient care by the oral health care team at different times of the patient’s life	72	72	29	2	–	12
	(38.5%)	(38.5%)	(15.5%)	(1.1%)		(6.4%)
31—Cleanliness and organisation of the clinic	61	87	31	6	–	2
	(32.6%)	(46.5%)	(16.6%)	(3.2%)		(1.0%)
32—Respect for the principles of infection control in dental practice	105	69	10	1	–	2
	(56.1%)	(36.9%)	(5.3%)	(0.5%)		(1.0%)
33—Supply of inputs and materials for the execution of satisfactory dental care	18	128	34	5	–	2
	(9.6%)	(68.4%)	(18.2%)	(2.7%)		(1.0%)
34—Using the clinical protocol in the activities of primary care	127	58	1	–	–	1
	(67.9%)	(31%)	(0.5%)			(0.5%)
35—Sufficient number of dentists to meet the service demands	31	34	30	21	67	3
	(16.6%)	(18.2%)	(16.0%)	(11.8%)	(35.8%)	(1.6%)
36—Knowledge of the major health problems of the community as well as help in resolving them	48	67	57	8	2	5
	(25.7%)	(35.8%)	(30.5%)	(4.3%)	(1.1%)	(2.7%)
37—Improvements to care and services provided to the population through courses and training for the oral health care team.	71	65	36	12	1	2
	(38.0%)	(34.8%)	(19.3%)	(6.4%)	(0.5%)	(1.1%)
38—Satisfaction with oral health care performance	54	112	16	2	–	3
	(28.9%)	(59.9%)	(8.6%)	(1.1%)		(1.6%)

## 3. Results

For most items (79.0%), professionals’ perceptions about comprehensiveness were overwhelmingly positive. When we stratified the analysis by the domains that make up the comprehensiveness of care, we found that the perceptions of professionals continued to be largely positive for all three domains: 77.3% for items that make up patient welcoming, 75.0% for the items that make up bonding and 87.5% items which make up quality of care. The five items (22.7%) in the patient welcoming domain about which the perception was less favourable were those about the participation of patients in health promotion activities in the waiting room; knowledge of other health care professionals about oral health care problems of the population of the region; systematic home visits by the oral health care team; planning patient care with the aid of other health professionals; and training of auxiliary team staff by the dentist.

The two items (25.0%) in the bonding domain for which the perception was less favourable were those about the return of the patient from specialised care with a written referral prepared by the specialist and permission for continuity of care in the flow between primary and specialised care. Lastly, the item in the quality of care domain for which the perception was less favourable (12.5%) concerned the sufficiency of the number of dentists to meet the service demands.

## 4. Discussion

The instrument aimed to assess, in addition to dental care in primary care, the knowledge and attitudes of dentists regarding the comprehensiveness of care and its domains. Dentists’ perception of the concept of comprehensiveness was largely positive. These results may reflect positively on the services because comprehensiveness of care is often used as a quality indicator of PHC [3,9,17], and it is considered an important principle in health systems around the world, such as in the Brazilian and Canadian systems [16,18]. In PHC, the concept is applied to the mandate to resolve and administer care for the most prevalent health conditions undifferentiated by sex, disease or age, and it has a second meaning that refers to the bio-psycho-social or whole-person approach, which sees the patient within a specific social context [1,6,8,9,19]. The understanding of this concept is very important for the satisfactory performance of PHC, and these dentists have demonstrated awareness about it for most of the domains. 

All of the dentists who made up the study sample were encouraged by the city hall of Belo Horizonte to do a specialisation course in family health care funded by the Brazilian health system. The training policy of the local government for human resources in healthcare may have contributed positively to the findings of the study. The advances in health systems and medical and dental knowledge, as well as the introduction of team-based and holistic, multifaceted patient-centred care, mean that improvements in population health and welfare increasingly depend on the renovation and maintenance of technical capacity among the healthcare workforce [10,11,20]. 

Stratifying the analysis by the domains that comprise the comprehensiveness of care, most of the issues that were less favourably perceived by the dentists in the patient welcoming domain are related to actions for human resources for health. The less favourable perceptions of the items “knowledge of other health care professionals about oral health care problems of the population of the region” and “planning patient care with the aid of other health professionals” imply a lack of integration between the oral health care team and the other health professionals who work in the same clinic. Historically, oral healthcare has been offered separately from other components integrated into general care [21]. Nevertheless, the association between systemic health and oral health demonstrates that collaborations among oral health professionals and other health professionals will be necessary for adequately addressing both the oral health care and the general health care of patients receiving healthcare services [19,22]. According to the literature, some oral diseases such as periodontal disease may be causes or risk factors for several systemic diseases such as diabetes, stroke, cardiovascular disease and atherosclerosis [23,24]. Because of these issues, collaboration amongst health professionals from both areas is paramount to minimise disparities between care and to ensure that the patient is the focus of an integrated approach to health. [25].

The observation of a less favourable perception of “training of auxiliary staff team by the dentist” suggests the need to consider that in team-based work, providing care in accordance with the guidelines set out in a high standard of quality workforce' necessitates engaging in new approaches to and processes in workforce planning [20]. These dentists seem to have a limited view about this approach. We need to remove the barriers for the development of efficient team-based work and to reinforce the processes of workforce planning; the guidelines that sustain the adequacy and quality of the future workforce can be constructed in line with this view [20,26,27].

In the domain of bonding, the dentists showed less favourable perceptions of items that described the performance and relationship between primary and secondary dental care. Many factors such as lack of a protocol for the construction of referral guidelines between primary and secondary dental care, deficiency in access to/availability of secondary care and lack of co-ordination between primary and secondary dental care may be considered in this context. Possible solutions include coordination to make services complementary and increasing capacity within both levels of care; however, we must note that the issues with the interface between primary and secondary dental care are complex and diverse. Individual measures are therefore likely to be partial and may themselves be complex in execution [28,29]. 

In the last domain, quality of care, the participants was concerned about an insufficient number of dentists to meet the service demands. The less favourable perception of this aspect shows that the number of dentists in the study is likely insufficient to care for the high volume of patients who seek health services daily. According to the literature, to obtain satisfactory performance of health services, the dental workforce should be adequate in quantity and skills to address the demand for dental care. Healthcare delivery is shaped by patients, professionals and managers. Adequate working conditions for healthcare stakeholders should be considered in any effort to provide care to a population; otherwise, the quality of care offered will be compromised [30,31].

Some limitations were faced during the development of the research. Although we used an instrument that has been validated to collect our data, we must consider that the findings for the city of Belo Horizonte do not represent all of Brazil [32]. We recommend future research with samples from different parts of the country because Brazil is a country of continental dimensions, and the perception of comprehensiveness of care by a professional may differ according to the diverse realities in which s/he operates. Furthermore, it is important to consider that this study is descriptive in nature, and it was not our aim at this time to identify and discuss the causes of the observed perceptions of the concept of comprehensiveness of care and its domains. In future research, we may apply the instrument to other samples in Brazil from different contexts, and further, we may apply the instrument to respondents from other countries, as the items may need to be validated in other languages. The analysis is also important for each group involved in health care delivery, so further research is needed to analyse and compare health services from different perspectives. [32,33]. On the other hand, a large portion of the Brazilian and world population lives in urban areas and in large cities. Thus, these results could be useful for populations and health systems similar to those in this study.

The descriptive analysis of a scenario or specific situation is a research focus that requires a large amount of effort, but it ultimately enhances the quality of the data collected [32,33]. In this study, human resources management, the dental workforce and the general population could well benefit from the findings. Most current approaches to assessing the quality of care are based on models developed over fifty years ago. The World Health Organization stated in a report [1] that with the growing importance of new experiences, person-focused appraisals should be prioritised over disease-focused appraisals. In this direction, approaches such as comprehensiveness should be studied more and explored more thoroughly so that health systems can benefit from the applicability of its concepts. These key features are increasingly recognised as having an impact on the way health services are delivered, and this study is in line with this statement [1,2,3,9,10,11,12,13].

## 5. Conclusions

Identifying and exploring the perceptions of dentists about the characteristics of comprehensiveness are among the main approaches to be considered in the assessment of primary care offered to the population by the Brazilian health system. The results suggest that the dentists in this study are aware of the importance of comprehensiveness for dental practices in primary care, and they also indicate that the concept should be strengthened in some specific areas. Our findings might be useful in further understanding human resources issues in health care and how the perceptions of the dentists can contribute to improvements in primary dental care. In future research, the perceptions of dentists from other parts of Brazil and from different nationalities can be studied to enrich and strengthen the data obtained here.
